# Attention Deficit Hyperactivity Disorder: Association With Obesity and Eating Disorders

**DOI:** 10.7759/cureus.12085

**Published:** 2020-12-14

**Authors:** Prithvi Ravi, Safeera Khan

**Affiliations:** 1 Psychiatry, California Institute of Behavioral Neurosciences & Psychology, Fairfield, USA; 2 Internal Medicine, California Institute of Behavioral Neurosciences & Psychology, Fairfield, USA

**Keywords:** adhd, obesity, overweight, eating disorders, psychostimulants, amphetamines, methylphenidate

## Abstract

Attention deficit hyperactivity disorder (ADHD) is one of the most common treatable psychiatric illnesses that affect all age groups from children to adults. Most commonly it is diagnosed in childhood or during teenage years. It can affect the mental and physical health of an individual and disrupt normal academic, career, and social functioning. The quality of life of the individual is affected; thus if diagnosed and treated, the results are good. Obesity and eating disorders are one of the comorbidities associated with ADHD and can lead to various other health problems. This study was done to find out the association between ADHD, obesity, eating disorders, and the effect of medication. We collected data from various studies through multiple electronic databases such as PubMed and Google Scholar. We found 8610 relevant articles and finally narrowed it down to 30 using various criteria. An association was found between ADHD, obesity, and eating disorders, although the mechanism linking ADHD, obesity, and eating disorders still remains unclear according to most studies. Some studies say ADHD medication helps in losing gained weight; some say they do not affect the weight.

## Introduction and background

In today’s world, psychiatric conditions have to be dealt with extreme importance as factors like stress and lifestyle can lead to mental health problems, which in turn can cause various systemic illnesses. These psychiatric conditions are both common in children and adults. Common classification of psychiatric conditions includes anxiety disorders, mood disorders, and personality disorders. Some psychiatric conditions present early in childhood and have the best outcome if diagnosed and treated early. Various studies have been done linking psychiatric conditions with each other, however not many studies have been done linking psychiatric conditions with systemic disorders.

Attention deficit hyperactivity disorder (ADHD) is one of the major psychiatric issues that affect all age groups including children, adolescents, and adults all over the world [1]. ADHD can be defined as a mental health disorder that causes the person to be hyperactive, impulsive, and have trouble paying attention to a given task. Most often, people with ADHD are unable to sit still and are excessively fidgety. ADHD can be classified as inattentive, hyperactive-impulsive, or combined [1]. Studies show the prevalence exceeds 5% in school-going children [2,3]. The onset of up to 65% of adults who have ADHD has been in childhood [2]. A lesser percentage of adults are diagnosed with new onset ADHD during adulthood. ADHD in adults has a prevalence of about ~2.5% [2]. Thus crucial academic years may be affected if ADHD is not diagnosed and managed appropriately. ADHD can be a risk factor for somatic diseases and vice versa [4].

Obesity is a major health issue that affects all socioeconomic classes across the world [5]. In today’s world, people do not have the time to prepare and consume wholesome healthy food and hence consume fast food that is cheap, quick, and easily available. Over the years, obesity has overtaken other health conditions that have a high level of morbidity and mortality. Worldwide, obesity-related medical expenditure is also expected to rise in the future [5]. Obesity increases the risk of other illnesses like hypertension, type 2 diabetes mellitus, sleep apnea, gallbladder disease, high cholesterol, breathing disorders, joint problems, musculoskeletal discomfort, fatty liver, and gastroesophageal reflux disease [3,6]. Obesity can also cause mental problems like depression and anxiety.

There have been various studies that show there is a relationship between ADHD and obesity [5,7]. The symptoms of ADHD might be the cause of the patient to have erratic eating habits that lead to them putting on weight and becoming obese [5,7-9]. Following treatment of ADHD with psychostimulants like methylphenidate, the symptoms of ADHD, such as impulsiveness, are reduced, and the patient becomes calmer and better organized [5,9]. Studies show patients without medical management of ADHD have a higher body weight when compared to patients who are on medical management [10]. Ultimately, the patient gains less weight and also has a decrease in appetite due to one of the most common side effects of methylphenidate: suppression of appetite [9,11].

There are various hypotheses explaining the relationship between ADHD and obesity. Studies have shown that obese individuals can present with ADHD, while individuals with ADHD can be diagnosed with obesity [12]. Some studies state that ADHD causes obesity due to impulsiveness and lack of organization [1,10,12]. Other studies show that obese individuals normally have sleep disturbances and disorders leading to ADHD [1,10,12].

Thus, this study is aimed to link the connection between ADHD, obesity, eating disorders, and how medical management of ADHD alters the course of the above comorbid conditions.

## Review

Method

We collected data through electronic databases: PubMed and Google Scholar. The data was collected in September 2020. The keywords used were “ADHD,” “obesity,” “overweight,” “eating disorders,” “psychostimulants,” “amphetamines,” “methylphenidate,” both separately and in combination with each other. We found 8610 articles with the help of the above keywords. The articles were screened by going through the abstracts in comparison to our research question. Inclusion and exclusion criteria were applied, and articles were further narrowed down to the most relevant and updated ones. The inclusion criteria were studies conducted on humans, studies in English, and studies in the last 10 years, relevant to our topic and research questions, peer-reviewed and full texts, or abstracts. The exclusion criteria were gray literature, letter to the editor, editorials, duplicates, overlapping studies, and animal studies.

Results

A total number of 8610 studies were identified from the databases searched. The inclusion criteria were of studies conducted on humans, studies in English, and studies in the last 10 years, relevant to our topic and research questions, peer reviewed and full texts, or abstracts. Based on the inclusion and exclusion criteria, articles were reduced to 355. The articles were screened, and the most relevant and updated studies were further shortlisted to 30. A total of 30 shortlisted articles were studied and analyzed [1-30].

Discussion

ADHD and Obesity

Obesity is a common risk factor associated with ADHD. Various studies have linked the association between ADHD and obesity. Children diagnosed with ADHD have an increased risk of developing obesity. A study conducted by Goulardins et al. about the relationship between ADHD and obesity suggests that obesity is a common risk factor for ADHD, suggesting that people with ADHD can develop obesity [13]. Many other studies suggest the same association between ADHD and obesity [14-16]. Hanć et al. found the same association between ADHD and obesity; however, the body mass index (BMI) was much lower in children below six years of age with ADHD and gradually increased over the year with development [17].

Impulsivity, which is the main symptom of ADHD, causes weight gain, thus causing obesity. Responses to food cues cause impulsivity, thus causing obesity. Matheson et al. suggested that the main cause of obesity in ADHD is impulsivity [18]. The study also suggested that an increase in BMI leads to decreased food cues inhibition, leading to obesity [18]. Various studies have found many mechanisms by which ADHD and obesity are associated; however, the exact mechanism explaining this association remains unclear. One of the explanations for this association could be how ADHD causes circadian rhythm problems. The circadian rhythm controls sleep and eating patterns; hence a disturbance in this rhythm can cause unstable eating patterns leading to obesity. Vogel et al. supports the above mechanism in their research [19]. Another study by Lundahl et al. also suggests the association between ADHD and circadian rhythm by portraying sleep disturbances in children with ADHD [20]. A study compared the physical activity and screen time of children with ADHD (six to 18 years) and children without and had lower physical activity and higher screen time, which puts them at a higher risk of developing obesity [21,22].

Some studies say gender has a role in determining the association between ADHD and obesity. A cohort study by Castaneda et al. consisting of 1001 subjects showed that female patients with ADHD were associated with childhood and young adulthood obesity. The strongest association was in 10 and 12 years in female children [22]. However, the same study did find a connection between ADHD and obesity in male patients [22]. Hormonal factors influence the connection between ADHD and obesity in females [22]. Males tend to be more hyperactive than females; thus, they tend to lose weight [22]. This study's main limitation was that the height and weight measurements were taken from retrospective record reviews recorded during a clinic visit as they may not be very accurate [22].

Many psychosocial risk factors have been noticed to cause obesity and ADHD. These include physical abuse, parental divorce, prolonged separation of parents, and stress. The research by Pauli-Pott et al. suggests that psychosocial risk factors can cause obesity and ADHD [23]. Another explanation for the association between ADHD and obesity could be chromosomal abnormalities. ADHD in individuals with certain alleles such as rs206936 and rs6497416 are at risk of developing obesity. Another study suggests that chromosomal abnormalities can cause obesity in patients with ADHD [18] (Table 1).

**Table 1:** Summarizing studies showing the association between ADHD and obesity ADHD, Attention deficit-hyperactivity disorder.

S. No.	Author	Year of publication	Purpose of the study	Type of study	Number of individuals	Result/Conclusion
1	Tandon et al. [21]	2019	To compare the physical activity and sleep of children with ADHD and children without ADHD	Cross-sectional study	N/A	Children with ADHD did not get enough physical activity and sleep.
2	Bowling et al. [15]	2018	To find out the association between childhood fat index and how severe are the symptoms of ADHD	Meta-analysis	3903	The more severe the symptoms of ADHD, the higher is the fat mass.
3	Matheson et al. [18]	2018	To determine if childhood behavior plays a role in increasing the risk of developing obesity	Literature review	N/A	Symptoms of ADHD in children predict the weight they put on overtime.
4	Chen et al. [14]	2017	To determine the association between excess weight in males and ADHD with their siblings	Meta-analysis	995,972	ADHD and overweight/obesity share familial risk factors.
5	Pauli-Pott et al. [23]	2017	To analyze whether the psychosocial risks explain the association between increased body weight and ADHD symptoms	Meta-analysis	360	A bad childhood may play a role in the processes that lead to the combined ADHD-overweight phenotype.
6	Castaneda et al. [22]	2016	To find out the rates of obesity during childhood and young adulthood in ADHD patients	Cohort study	1001	Females with ADHD in childhood and young adulthood are associated with obesity.
7	Goulardins et al. [13]	2016	To find out the relationship between motor performances, symptoms of ADHD, and fat in children	Cross-sectional study	189	The number of overweight ADHD children was lower than the number of overweight control children and overweight motor impairment children.
8	Lundahl et al. [20]	2016	To find out if sleep problems play a role in the connection between ADHD and obesity	Traditional review	N/A	Disturbed sleep plays a role in the connection between ADHD and obesity.
9	Vogel et al. [19]	2015	To find out if a disturbance in circadian rhythm is involved in a process connecting ADHD symptoms to obesity	Case-control study	470	Circadian rhythm disturbance is involved in the process connecting ADHD symptoms to obesity.
10	Hanć et al. [17]	2015	To determine if there are variations in body size between preschool boys with and without ADHD	Cross-sectional study	420	Boys in preschool with ADHD in the age group of two to six years are more likely to have a lower weight than their peers. But later on during development, they are shorter and more commonly overweight than boys without ADHD.
11	Cortese et al. [16]	2013	Comparison of BMI and rates of obesity in adult men with and without childhood ADHD	Meta-analysis	207	Adults would be at an increased risk of obesity if they were diagnosed with ADHD in their childhood.

ADHD, Eating Disorders, and the Effect of Medication

ADHD and eating disorders have recently been closely linked together, showing an association between these two. However, the reasons for this connection remain unclear. For instance, researchers believe that the same underlying factors cause ADHD and eating disorders. For example, eating disorders might result from trying to manage ADHD stress. ADHD, obesity, and eating disorders have shared neuropsychological dysfunction. A study by Van der Oord et al. supports this statement by finding an association between obesity and ADHD. Obesity, not alone, but with binge eating, is associated with ADHD [24]. Another study by Egbert et al. also found the same association between ADHD and obesity and its relation to eating disorders [25]. The impulsivity and inattentiveness in ADHD promote bulimic symptoms, whereas inattention and hyperactivity are associated with craving. The association of ADHD with obesity could also be due to the distorted sense of self-awareness and body image. Studies show that inattentive and impulsive behavior specific in patients with ADHD led to impulsive eating behavior and loss of control, leading to obesity [26,27]. A study by Quesada et al. also suggests that patients with ADHD have no motivation to participate in physical activity, thus leading to obesity [26].

The impulsiveness in patients with ADHD leading to eating disorders was found to be more common in obese females when compared to males. A study by Nazar et al. also suggested that the eating disorders associated with ADHD are more commonly noticed in females [27]. The study done by Quesada et al. also found the same gender association and suggested that another reason for the more common obesity in female patients with ADHD could be lack of physical activity [26].

Patients with ADHD are also highly likely to experience mood swings and depression. Depression is commonly known to cause eating disorders. Thus, patients with ADHD who suffer from depression have eating disorders. Tong et al. did a study and found the link between ADHD and depression, causing eating disorders [28].

A deficiency in the two neurotransmitters, norepinephrine and dopamine, can lead to an increased desire to overeat, poor self-esteem, inability to follow a meal plan, and inability to accurately judge portion size, and inability to put an end to binge eating. ADHD was the first disorder found to be the result of the deficiency of the above neurotransmitters. ADHD is also found to respond to medications that correct the deficiency of these neurotransmitters. For example, stimulant drugs like amphetamine and methylphenidate are the treatment of choice for ADHD. Simultaneously, these medications can make the patient feel less hungry and make their bodies burn calories faster than usual. These medications are used to help people lose weight and treat binge eating [26]. Granato et al. suggests that methylphenidate use in patients who have been diagnosed with ADHD and obesity is relevant not only for controlling symptoms of ADHD but also for improving these individuals' nutritional status [12]. Few more studies also support that ADHD medication can improve the BMI of the patient [9,29].

However, in some studies, a higher frequency of obesity was found associated with methylphenidate use; this may be due to the change in eating habits. Patients taking drugs can eat more frequently at night when the drugs no longer affect the symptoms' level and suppress appetite. A study done by Hanć supports the fact that methylphenidate does not help in weight loss with ADHD [30]. Another study by Racicka et al. also suggested that the ADHD-obesity relationship remains significant regardless of whether the children underwent medical treatment or not [10] (refer to Table 2).

**Table 2:** Summarizing the association of ADHD with eating disorders leading to obesity and the effect of treatment ADHD, Attention deficit-hyperactivity disorder; BMI, body mass index.

S. No.	Author	Year of publication	Purpose of the study	Type of study	Number of subjects	Result/Conclusion
1	Racicka et al. [10]	2018	To determine the ubiquity of overweight and obesity in children and teenagers with ADHD considering pharmacological treatment and comorbidities	Meta-analysis	408	BMI, the proportion of overweight, and obesity were greater in patients with ADHD compared with the general population.
2	Granato et al. [12]	2018	To find out the associations between ADHD treatment and patient nutritional status and height	Cohort study	252	Methylphenidate is associated with a reduction in BMI in patients with ADHD who are overweight or obese.
3	Vander Oord et al. [24]	2018	To find out the association between binge eating, obesity, and ADHD	Case control	64	The mechanism reported in ADHD is due to obesity along with binge eating.
4	Quesada et al. [26]	2018	To determine studies finding out the role of eating behavior linking ADHD and obesity in children and teenagers	Systematic review	N/A	The inattentiveness and impulsiveness that characterize ADHD could contribute to disordered eating behaviors.
5	Hanć et al. [30]	2018	To find out the effect of pharmacological treatment on obesity in ADHD patients	Literature review	N/A	Medication plays a role in the association between ADHD and obesity.
6	Bowling et al. [9]	2017	To find out the connections between ADHD, stimulant use, and change in BMI in children as well as differences in diet and exercise that may mediate the connection between the use of stimulants and change in BMI	Cohort study	8250	The use of stimulants predicted greater BMI trajectory between fifth and eighth standard class but did not affect dietary or exercise patterns.
7	Tong et al. [28]	2017	To find out the associations between ADHD, abnormal eating, and BMI	Cross-sectional study	785	There is a significant relationship between ADHD and eating disorders; however, there is no significant association between ADHD, eating disorders, and BMI.
8	Egbert et al. [25]	2017	To find out the association between ADHD and maladaptive eating taking into consideration loss of control	Meta-analysis	385	ADHD symptoms appear to be associated with overeating in youth both with and without loss of control.
9	Nazar et al. [27]	2016	To find out the proportion of ADHD in obese women seeking treatment and its connection with binge eating and bulimic symptoms	Cross-sectional study	155	Obese individuals who have ADHD may be at a higher risk for more severe erratic eating patterns.
10	Cortese et al. [29]	2014	To examine the clinical implications of treating ADHD in individuals with obesity	Literature review	N/A	Treatment of ADHD might characteristically increase the effectiveness of weight management strategies.

Limitations

The mechanism involving the association between ADHD, obesity, eating disorders, and the effect of medication still remains unclear. Various studies also contradict each other of the effect of ADHD medication on obesity. The other limitations of this traditional review were that only studies from the last 10 years were included, and only human studies were included.

## Conclusions

This study was done to determine how ADHD, obesity, eating disorders, and ADHD medication are connected and how they affect each other. Children diagnosed with ADHD have an increased risk of developing obesity. Impulsivity is the main symptom of ADHD that causes patients to develop obesity. The mechanism involved in linking ADHD and obesity remains unclear. Children diagnosed with ADHD are found to have lesser physical activity and more screen time; thus, having a sedentary lifestyle increases obesity. Gender also plays a role in this association. For example, males tend to be more hyperactive than females, hence losing the gained weight more easily than females. Therefore, females have a higher risk of developing obesity. Various psychosocial factors and a bad childhood also play a role in developing obesity as depressed individuals have erratic eating patterns leading to obesity. The impulsive and inattentive behavior in individuals with ADHD causes them to have erratic eating patterns, leading to obesity. Individuals are more likely to suffer from depression, hence more likely to gain weight. Further research is needed to find out the exact mechanism. More studies involving patients of various age groups, gender, and medication effects also need to be addressed.

## References

[1] Güngör S, Celiloğlu ÖS, Raif SG, Özcan ÖÖ, Selimoğlu MA (2016). Malnutrition and obesity in children with ADHD. J Atten Disord.

[2] Cortese S, Tessari L (2017). Attention-deficit/hyperactivity disorder (ADHD) and obesity: update 2016. Curr Psychiatry Rep.

[3] Hanć T, Cortese S (2018). Attention deficit/hyperactivity-disorder and obesity: a review and model of current hypotheses explaining their comorbidity. Neurosci Biobehav Rev.

[4] Chen Q, Hartman CA, Kuja-Halkola R, Faraone SV, Almqvist C, Larsson H (2019). Attention-deficit/hyperactivity disorder and clinically diagnosed obesity in adolescence and young adulthood: a register-based study in Sweden. Psychol Med.

[5] Cortese S, Moreira-Maia CR, St Fleur D, Morcillo-Peñalver C, Rohde LA, Faraone SV (2016). Association between ADHD and obesity: a systematic review and meta-analysis. Am J Psychiatry.

[6] Cook BG, Li D, Heinrich KM (2015). Obesity, physical activity, and sedentary behavior of youth with learning disabilities and ADHD. J Learn Disabil.

[7] Martins-Silva T, Vaz JDS, Hutz MH (2019). Assessing causality in the association between attention-deficit/hyperactivity disorder and obesity: a Mendelian randomization study. Int J Obes (Lond).

[8] Brunault P, Frammery J, Montaudon P (2019). Adulthood and childhood ADHD in patients consulting for obesity is associated with food addiction and binge eating, but not sleep apnea syndrome. Appetite.

[9] Bowling A, Davison K, Haneuse S, Beardslee W, Miller DP (2017). ADHD medication, dietary patterns, physical activity, and BMI in children: a longitudinal analysis of the ECLS-K study. Obesity (Silver Spring).

[10] Racicka E, Hanć T, Giertuga K, Bryńska A, Wolańczyk T (2018). Prevalence of overweight and obesity in children and adolescents with ADHD: the significance of comorbidities and pharmacotherapy. J Atten Disord.

[11] Nigg JT, Johnstone JM, Musser ED, Long HG, Willoughby MT, Shannon J (2016). Attention-deficit/hyperactivity disorder (ADHD) and being overweight/obesity: new data and meta-analysis. Clin Psychol Rev.

[12] Granato MF, Ferraro AA, Lellis DM, Casella EB (2018). Associations between attention-deficit hyperactivity disorder (ADHD) treatment and patient nutritional status and height. Behav Neurol.

[13] Goulardins JB, Rigoli D, Piek JP (2016). The relationship between motor skills, ADHD symptoms, and childhood body weight. Res Dev Disabil.

[14] Chen Q, Kuja-Halkola R, Sjölander A (2017). Shared familial risk factors between attention-deficit/hyperactivity disorder and overweight/obesity - a population-based familial coaggregation study in Sweden. J Child Psychol Psychiatry.

[15] Bowling AB, Tiemeier HW, Jaddoe VWV, Barker ED, Jansen PW (2018). ADHD symptoms and body composition changes in childhood: a longitudinal study evaluating directionality of associations. Pediatr Obes.

[16] Cortese S, Olazagasti MAR, Klein RG, Castellanos FX, Proal E, Mannuzza S (2013). Obesity in men with childhood ADHD: a 33-year controlled, prospective, follow-up study. Pediatrics.

[17] Hanć T, Słopień A, Wolańczyk T (2015). Attention-deficit/hyperactivity disorder is related to decreased weight in the preschool period and to increased rate of overweight in school-age boys. J Child Adolesc Psychopharmacol.

[18] Matheson BE, Eichen DM (2018). A Review of childhood behavioral problems and disorders in the development of obesity: attention deficit/hyperactivity disorder, autism spectrum disorder, and beyond. Curr Obes Rep.

[19] Vogel SW, Bijlenga D, Tanke M (2015). Circadian rhythm disruption as a link between attention-deficit/hyperactivity disorder and obesity?. J Psychosom Res.

[20] Lundahl A, Nelson TD (2016). Attention deficit hyperactivity disorder symptomatology and pediatric obesity: psychopathology or sleep deprivation?. J Health Psychol.

[21] Tandon PS, Sasser T, Gonzalez ES, Whitlock KB, Christakis DA, Stein MA (2019). Physical activity, screen time, and sleep in children with ADHD. J Phys Act Health.

[22] Castaneda RL, Kumar S, Voigt RG (2016). Childhood attention-deficit/hyperactivity disorder, sex, and obesity: a longitudinal population-based study. Mayo Clin Proc.

[23] Pauli-Pott U, Reinhardt A, Bagus E, Wollenberg B, Schroer A, Heinzel-Gutenbrunner M, Becker K (2017). Psychosocial risk factors underlie the link between attention deficit hyperactivity symptoms and overweight at school entry. Eur Child Adolesc Psychiatry.

[24] Van der Oord S, Braet C, Cortese S, Claes L (2018). Testing the dual pathway model of ADHD in obesity: a pilot study. Eat Weight Disord.

[25] Egbert AH, Wilfley DE, Eddy KT (2018). Attention-deficit/hyperactivity disorder symptoms are associated with overeating with and without loss of control in youth with overweight/obesity. Child Obes.

[26] Quesada D, Ahmed NU, Fennie KP, Gollub EL, Ibrahimou B (2018). A review: associations between attention-deficit/hyperactivity disorder, physical activity, medication use, eating behaviors and obesity in children and adolescents. Arch Psychiatr Nurs.

[27] Nazar BP, de Sousa Pinna CM, Suwwan R (2016). ADHD rate in obese women with binge eating and behaviors from a weight-loss clinic. J Atten Disord.

[28] Tong L, Shi H, Li X (2017). Associations among ADHD, abnormal eating and overweight in a non-clinical sample of Asian children. Sci Rep.

[29] Cortese S, Castellanos FX (2014). The relationship between ADHD and obesity: implications for therapy. Expert Rev Neurother.

[30] Hanć T (2018). ADHD as a risk factor for obesity. Current state of research. Psychiatr Pol.

